# Demographic-Based Content Analysis of Web-Based Health-Related Social Media

**DOI:** 10.2196/jmir.5327

**Published:** 2016-06-13

**Authors:** Shouq A Sadah, Moloud Shahbazi, Matthew T Wiley, Vagelis Hristidis

**Affiliations:** ^1^ University of California, Riverside Department of Computer Science and Engineering Riverside, CA United States; ^2^ SmartDocFinder LLC Riverside, CA United States

**Keywords:** Web-based social media, demographics, content analysis, health forums, drug reviews

## Abstract

**Background:**

An increasing number of patients from diverse demographic groups share and search for health-related information on Web-based social media. However, little is known about the content of the posted information with respect to the users’ demographics.

**Objective:**

The aims of this study were to analyze the content of Web-based health-related social media based on users’ demographics to identify which health topics are discussed in which social media by which demographic groups and to help guide educational and research activities.

**Methods:**

We analyze 3 different types of health-related social media: (1) general Web-based social networks Twitter and Google+; (2) drug review websites; and (3) health Web forums, with a total of about 6 million users and 20 million posts. We analyzed the content of these posts based on the demographic group of their authors, in terms of sentiment and emotion, top distinctive terms, and top medical concepts.

**Results:**

The results of this study are: (1) Pregnancy is the dominant topic for female users in drug review websites and health Web forums, whereas for male users, it is cardiac problems, HIV, and back pain, but this is not the case for Twitter; (2) younger users (0-17 years) mainly talk about attention-deficit hyperactivity disorder (ADHD) and depression-related drugs, users aged 35-44 years discuss about multiple sclerosis (MS) drugs, and middle-aged users (45-64 years) talk about alcohol and smoking; (3) users from the Northeast United States talk about physical disorders, whereas users from the West United States talk about mental disorders and addictive behaviors; (4) Users with higher writing level express less anger in their posts.

**Conclusion:**

We studied the popular topics and the sentiment based on users' demographics in Web-based health-related social media. Our results provide valuable information, which can help create targeted and effective educational campaigns and guide experts to reach the right users on Web-based social chatter.

## Introduction

As Web-based social media are growing in popularity, the number of people who share their experiences or ask for support in health-related social media has also increased [1]. Fox and Jones have found that 41% of e-patients have read someone else’s commentary or experience about health on a Web-based news group, website, or blog [2]. Kane et al [3] reported that more than 60 million Americans read or contribute to Health 2.0 apps, in which they consider these apps as their first source when gathering data and opinions. About 40% of Americans doubt a professional opinion when it conflicted with what they form from Web-based health social media [3].

One of the key benefits of health-related Web-based social media reported by researchers is the increased access to information to various demographic groups, regardless of age, education, income, or location [4]. However, previous work has mainly relied on user surveys to study the effect of the use of social media to health-related factors such as psychological distress [5]. In addition, previous work does not reveal granular information on what disorders or other health topics are mostly discussed in the Internet by each demographic group, which would allow health care providers to create targeted and effective educational campaigns.

In this work, we conducted the first, to our best knowledge, large-scale data-driven comparative analysis of the content of health-related social media across various demographic dimensions—gender, age, ethnicity, location, and writing level. For each demographic group, we study the content of the posts across the following dimensions: sentiment, popular terms (keywords), and medical concepts (particularly disorders and drugs). Concepts refer to entries in the Unified Medical Language System (UMLS) vocabulary [6], whereas terms are just words from the posts’ text that may or may not belong to any UMLS concept. We report results for 3 types of social media: (1) general Web-Based Social Networks, namely Google+ and Twitter, (2) drug review websites, and (3) health Web forums. The selection of social media types was based on their popularity and on our study of the literature on health-related social content [7]. The objective of this study was to identify which health topics are discussed in which social media by which demographic groups, to better guide educational outreach and research activities.

### Related Work

#### Analysis of Health-Related Social Media

Different studies were established and conducted by researchers to study the effectiveness of Web-based social media in changing and improving the communication between providers and patients. Hackworth and Kunz [8] reported that 80% of Americans have searched the Internet for health-related information. Grajales et al [9] illustrated how, when, and why social media are used by health care sectors by conducting a narrative review of case studies, and they provided 4 recommendations that stakeholders may consider to engage with social media. Because analyzing the health-related content of social media is increased recently [10], Denecke and Nejdl [11] performed content analysis of medical concepts in different health-related social media sources. They presented a method to classify posts as informative or affective, and they found that doctors share health-related information, whereas patient and nurses are more likely to share personal experiences. Lu et al [12] analyzed the content of 3 disease-specific health communities including lung cancer, breast cancer, and diabetes and defined their relationship to 5 main informative topics: symptoms, complications, examination, drugs, and procedures. This study shows that examination is a hot topic for users with breast cancer, whereas symptoms are more likely to be discussed by users with lung cancer. Wiley et al [13] analyzed the content of drug-related chatter on various social media forums. The study demonstrates that Web-based social media's characteristics such as moderation affect the discussions in different ways including subjectivity and type of drugs discussed.

#### Measuring and Estimating Demographics of Users of Social Media

Krueger et al [14] studied the mortality attributable to low education level in the United States. They found people with less than high school degree have more mortality rate; thus, improving the US educational attainment could increase the survival in US population. A Pew research conducted in 2012 showed that white ethnicity represents 75% of social media websites users, where women in age group of 30-49 years participate more in these websites [15]. Another study by eMarkter found that Hispanic are more active in social media with 68.9% of them using social networks compared with 66.2% of total US population [16]. Mislove et al [17] estimated gender and ethnicity for Twitter users. The gender is estimated by using the reported first name and comparing it to the 1000 most popular first names reported by the US Social Security Administration, whereas ethnicity is estimated by using the reported last name and comparing it to the frequently occurring surnames reported by 2000 US Census. Using Mislove’s gender classifier, Mandel et al [18] analyzed the tweets related to Hurricane Irene. Liu et al [19] proposed Natural Language Processing (NLP) methods to extract the demographics (gender, age, ethnicity) of users of social posts. Anderson-Bill et al [20] recruited Web-health users to examine their demographics, behavioral, and psychosocial characteristics, and they found that Web-health users are more likely middle-aged, upper class, and well-educated women. Although the aforementioned work examined health-related social media and their content, none of them studied how different demographics use Web-based social media, which is studied in this work.

Sadah et al [21] studied how many users from each demographic group (by gender, age, ethnicity, location, and writing level) participate in various social media, but it did not study the content of the posts, which is the focus of this paper. Some of the key findings of that work are: (1) drug review websites and health Web forums are dominated by female users; (2) the participants of health-related social media are generally older with the exception of the 65+ years bracket; (3) Asian and black ethnic groups are underrepresented in drug review websites and health Web forums, and blacks are also underrepresented in health-related Web-based social networks; (4) users in areas with better access to health care participate more in Web-based health social media; and (5) the writing level of users in health social media is significantly lower than the reading level of the population.

## Methods

### Key Challenges

A key challenge is to estimate the demographic group, for example, gender, of a Web-based user when this information is not explicitly stated. Another challenge in this work is the extraction of medical concepts from social posts, given that existing tools such as MetaMap focus on biomedical text, which is generally generated by researchers or practitioners; therefore, we filtered out some misclassified concepts generated by this tool to work on health social media posts. Another challenge has been the time to extract the medical concepts from the social posts. In this paper, we process more than 20 million posts, which would take several months to parse on a single machine. For that, we have parallelized this into 10 machines that extracted all concepts in about 1 month. To extract popular terms for each demographic group, we use stemming to merge together terms with the similar root.

### Datasets

As summarized in Table 1, for general social networks, we chose Twitter and Google+ for their popularity and number of users (we did not include Facebook as it does not provide public data). For the other 2 types, we selected 3 different websites for each one to ensure diversity. More information about the sources including start and end date is available in Table A.1 and A.2 of Multimedia Appendix 1. Because Twitter and Google+ are general social networks, we filtered the posts using 276 representative health-related keywords as follows: (1) Drugs: from the most prescriptions dispensed from RxList.com, we selected the 200 most popular drugs [22]. By removing the variants of the same drug (eg, different milligram dosages), the final list of drugs contained 125 unique drug names. (2) Hashtags: from Twitter Hashtags, we selected 11 popular health-related Twitter hashtags such as #BCSM (Breast Cancer and Social Media). (3) Disorders: 81 popular disorders were selected such as AIDS and asthma. (4) Pharmaceuticals: the 12 largest pharmaceutical companies were selected such as Novartis. (5) Insurance: 44 of the biggest insurances were selected such as Aetna and Shield. The rationale of selecting the keywords was to cover as much as we can by including popular drugs and disorders, popular health-related hashtags in Twitter, and other related health keywords that can help increase the number of the posts related to health, similar to previous work on Twitter filtering [13,21]. A complete list of used keywords can be found in Table B.1 of Multimedia Appendix 1, and all terms’ frequencies for both sources can be found in Table B.2 of Multimedia Appendix 1.

**Table 1:** List of all used sources with their number of posts and with the available demographic attributes.

Dataset	No. of posts	Gender^a^	Age^a^	Ethnicity^a^	Location^a^	Writing level
TwitterHealth [23]	11,637,888	Gender classifier	NO	Ethnicity classifier	YES	Writing level classifier
Google+Health [24]	186,666	YES	YES	Ethnicity classifier	YES	Writing level classifier
Drugs.com [25]	74,461	Gender classifier	NO	NO	NO	Writing level classifier
DailyStrength/Treatments [26]	1,055,603	YES	YES	NO	YES	Writing level classifier
WebMD/Drugs [27]	122,040	YES	YES	NO	NO	Writing level classifier
Drugs.com/Answers [28]	320,118	Gender classifier	NO	NO	NO	Writing level classifier
DailyStrength/Forums [29]	5,948,877	YES	YES	NO	YES	Writing level classifier
WebMD [30]	1,128,629	Gender classifier	NO	NO	NO	Writing level classifier

^a^*NO* indicates that the demographic attribute is not provided by the source and no classifier is used due to low accuracy. *YES* indicates that the demographic attribute is provided by the source. More details on the demographic classifiers are available in the paper by Sadah et al [21].

**Figure 1:**
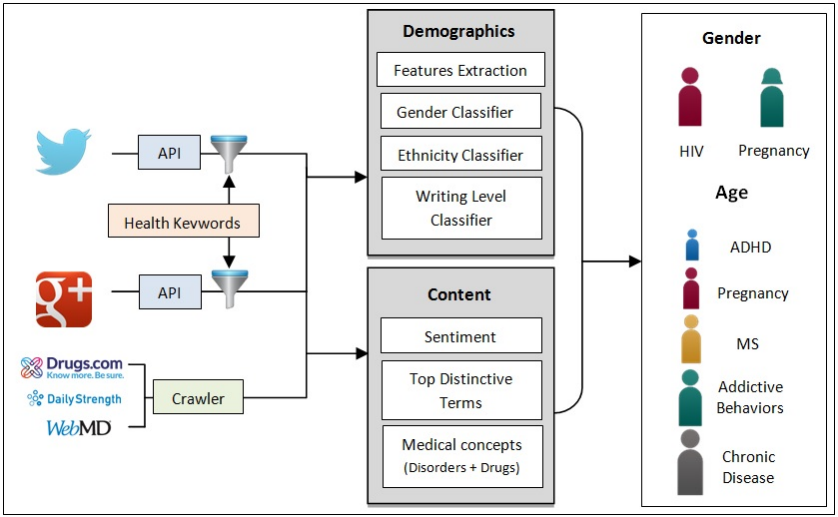
Overview of the data collection and analysis process.

Then, to filter out Twitter using the health-related keyword list, we used the Twitter streaming Application Program Interface (API) [31] to extract the relevant tweets for TwitterHealth. Google+Health posts were collected via the Google+ API [32], in which the health-related keyword list was used in the queries to obtain relevant posts for Google+. For the other drug review websites and health Web forums, we built a crawler for each website in Java using the Java library jsoup [33] for extracting and parsing hypertext markup language content. For each website, we crawled and collected the available data, including public user information, posts, disorders, conditions, keywords, tags, rating, and so forth. We emphasize that we do not collect or use any private data, and we only collected publicly available data in accordance with each site’s terms of use. Figure 1 shows the overall process of our analysis.

### Demographic Data Computation

The demographic data (age, gender, ethnicity, location, writing level) of users are extracted from the data or estimated through classifiers as discussed in the study by Sadah et al where more statistics of the collected posts such as dates and number of users are also reported [21]. As summarized in Table 1, gender attribute is either reported by the source or generated by a classifier that uses the first name to distinguish between male and female. Age and location, on the other hand, are used as they reported by the source; however, location was further processed to map user’s input into geolocations using Google API [32]. Because ethnicity is not reported in any source, we used a classifier that uses the last name to predict ethnicity for users in sources that provide the last name. For writing level, we measured each user writing level using a modified version of Flesch–Kincaid Grade Level [34].

### Sentiment and Emotion

To compute sentiment and emotion, we map each phrase in the post to a phrase from a sentiment lexicon. We use a sentiment lexicon, SentiWordNet [35], and an emotion lexicon, NRC word-emotion lexicon [36]. These 2 lexicons were selected owing to their effectiveness and popularity in previous studies [11,37,38] and because they cover complementary aspects. We use the SentiWordNet dictionary for sentiment, which assigns positive, negative, and objective score to each term where the sum of all 3 scores equals 1. Because SentiWordNet uses senses and part of speech, the Stanford CoreNLP Trigger [39] was used to tag each word with its part of speech tag. All words in posts and SentiWordNet were then stemmed to remove words variation. The longest possible match is then used to map each phrase in posts to a phrase from SentiWordNet, and after that, each post’s sentiment is calculated by averaging scores of all phrases. For each source, the total sentiment score for each demographic attribute is measured by averaging all posts' scores associated with that attribute and normalized by the number of posts of the attribute. For emotion, we use the NRC word-emotion lexicon, which measures anger–fear, trust–disgust, and anticipation–surprise.

### Top Distinctive Terms

The content of all sources was analyzed to get the top distinctive terms for each source. All posts are first filtered to remove stop words and then stemmed using Porter stemmer [40]. From these words, we considered only the ones that occur in at least 0.01% of the total number of posts that are annotated for a given demographic attribute value (or 30 if 0.01% is less than 30). That is, if less than 0.01% of posts from users who reported their gender contain a term, this term is not reported in either male or female group analysis. Then, for each demographic attribute value, that is, male, we normalized the number of occurrences for each term in that attribute value by the number of users posts in the same attribute value to get the frequency, for example:

Freqmale (headache)=No. of occurrences (headache) in male/No. of male posts

1

To get the top 10 distinctive terms for each demographic attribute, we then calculated the relative difference as follows:

RelDifmale (headache)=[Freqmale (headache) − AvgFreqgender (headache)] / AvgFreqgender (headache)

2

Where *AvgFreq*_gender_ (*headache*) is the average frequency of the word headache in all posts by male or female users. For example, *AvgFreq*_location_*(headache) =* [*Freq*_Northeast_(*headache*) *+ Freq*_Midwest_(*headache*) *+ Freq*_South_(*headache*) *+ Freq*_West_(*headache*)] /4. Finally, we only display health-related terms in each demographic group that have a relative difference greater than 0.1; that is, we decided to hide results with a difference of less than 10% from the average score, which we believe is intuitive.

### Medical Concepts

To annotate posts with corresponding medical concepts from the UMLS [41], the MetaMap tool [42] was used to represent each post as a set of medical concepts.

Because MetaMap was originally built to extract concepts from biomedical text generated by researchers or practitioners, it is not perfect to annotate social media posts [43]. Therefore, we manually removed some annotations that were misclassified by MetaMap as following: (1) we order generated concepts by their frequencies for each source systematically, (2) we analyze each phrase that was mapped for each concept, and (3) we delete the misclassified UMLS concepts from the results. For example, the letter “i” mapped to (immunologic factor) and word bad mapped to (organic chemical). Such mistakes were deleted from MetaMap annotations to improve accuracy. In UMLS, we have 15 semantic groups (eg, Disease or Anatomy), and each concept in UMLS is associated with one or more semantic types, where each semantic type belongs to 1 semantic group. In this part, we analyzed only 2 semantic groups including drugs and disorders, and we reported the top distinctive drugs and disorders for each demographics using the same threshold and method used in finding top distinctive terms (Equation 2).

## Results

In this section, we present our results for sentiment and emotion, top distinctive terms, and medical concepts by each demographic group. Two medical concept types were considered and reported to avoid less interesting results: disorders and drugs. For each demographic group, we show the top distinctive disorders and drugs using Equation 2 that have a relative difference more than 0.1. Some demographic attribute values are not reported owing to small number of users (age group (0-17) and (65+) in Google+Health), or demographic attribute is not reported by the source (all age groups in TwitterHealth), or because users talk about unrelated health topics (writing level (0-5) in TwitterHealth talk about astrology), or the relative difference (Equation 2) for the top findings is less than 0.1.

### Gender

In Table 2, we summarize the top distinctive (highest relative difference according to Equation 2) terms by gender; note that some demographic attributes such as female in Google+Health do not have distinctive terms. Because Twitter and Google+ are more news-based social media, many health posts share news in different areas including politics and sports—we excluded them to include health-related keywords only. Our first key finding is that male users in TwitterHealth tend to talk more about the reproductive system, tumor and AIDS, and health insurance, whereas female users talk about headache and emotion. In drug review websites and health Web forums, female users tend to talk more about pregnancy-related topics, whereas male users discuss pain drugs, cholesterol, and heart problems.

**Table 2:** Top 10 distinctive terms by gender.

Gender	TwitterHealth	Google+Health	Drugs	Forums
Male	Prostate, Gay, Testicular, Viagra, Tumor, AIDS, Obamacare, Marijuana, Medicare, Insurance	Pharmacology, Encephalomyelitis, Amphetamine, Pertussis, Fukushima, Pfizer, Novartis, Neutrophil, Biomed, Viagra	Wife, Oxycontin, Urine, Lisinopril, Cholesterol, Hydrocodone, Disc, Spinal, Libido, Diovan	HIV, Wife, Tinnitus, Gay, Cholesterol, Artery, AA (alcoholic anonymous), Valium, Cardiologist, Alcohol
Female	Cry, Migraine, Moody, Frown, Pound, Laugh, Nap, Eczema, Headache, Tension	N/A	Ovulation, IUI (intrauterine insemination), Pregnancy, Clomid (used to cause ovulation in women), IVF (in vitro fertilization), Pregnant, Birth, Boyfriend, BC (birth control), Fibromyalgia	Miscarry, PCOS (polycystic ovary syndrome), Endometriosis, Lupron, Uterus, Hysterectomy, Infertility, Ovarian, Rheumatologist, Progesterone

In Table 3, we summarize top distinctive disorders by gender. Male users in drug review websites mainly talk about back pain and blood pressure, whereas female users talk about pregnancy. In health Web forums and websites, male users discuss heart problems and panic topics, and female users talk more about skin disorders, headache, and chronic fatigue disorders. In TwitterHealth and Google+Health, top disorders discussed by male users can be classified as sexually transmitted diseases, including AIDS and herpes, whereas female in TwitterHealth as seen in the top distinctive terms discuss topics related to headache and feelings.

**Table 3:** Top 5 distinctive disorders by gender.

Gender	TwitterHealth	Google+Health	Drugs	Forums
Male	Acquired Immunodeficiency Syndrome (AIDS), HIV seropositivity, Cerebrovascular accident (stroke), Incised wound, Herpes NOS	Gonorrhea, Marijuana abuse, Sexually transmitted diseases, Malignant neoplasm of lung, Infantile neuroaxonal dystrophy	Low back pain, Dry cough, Blood pressure finding, Back pain, Diabetic	Atrial fibrillation, Codependency, Panic attacks, Diabetes, Marijuana abuse
Female	Migraine disorders, Emotional, Headache, Pain NOS adverse event, Asleep	Chronic fatigue syndrome	Gravidity; Endometriosis, site unspecified; Yeast infection; Fibromyalgia; Hot flushes	Dermatitis herpetiformis, familial; Lupus vulgaris; Lupus erythematosus, systemic; Fibromyalgia; Migraine disorders

Table 4 summarizes top distinctive drugs by gender. In drug review websites, the top drugs discussed by female users are related to pregnancy including birth control and ovulation stimulation, whereas male users talk mainly about drugs related to blood pressure. In health Web forums ,male users discuss depression-related drugs and alcohol topics. In TwitterHealth, not many distinctive drugs were found for female and male users, whereas in Google+Health, different drugs and chemicals were reported.

Sentiment and emotion were evaluated for all sources. Because the results look similar between gender groups, we summarize the results in Tables C.1 and C.2 of Multimedia Appendix 1.

**Table 4:** Top 5 distinctive drugs by gender.

Gender	TwitterHealth	Google+Health^a^	Drugs^a^	Forums^a^
Male	Viagra	Aldosterone, DC101 monoclonal antibody, Bicarbonates, Aspartame, Methamphetamine^1^	Low-density lipoproteins, Plavix, Bystolic^6^, Oxycodone, Opiates	Alcohols, Xanax^4^, Detox adjuvant^2^, Prozac^4^, Dietary lead
Female	Trivalent influenza vaccine	Thioctic acid, Detoxadjuvant^2^, Seroquel	Yaz^3^, Implanon^3^, Tamoxifen^2^, Estrogens, Clomid^3^	Plaquenil, Diamox, Topamax, Concerta, Synthroid

^a^Some of the drugs are coded to match the corresponding disorders they treat:^1^ADHD,^2^Cancer,^3^pregnancy,^4^depression,^5^MS,^6^BP, heart problem and cholesterol,^7^Diabetes.

### Age

Table 5 summarizes the top 10 distinctive terms for each age group. Generally, for younger groups (0-17 years), ADHD and skin problems are popular topics in drug review websites, whereas in health Web forums, they talk more about parents and homosexuality. For age groups of 18-34 years in drug review websites and health Web forums, the main topics discussed are related to relationships, pregnancy, or getting pregnant using simple intervention methods, or family members; whereas the same groups in Google+Health talk about different aspects including vitamins and sleep disorders. Age group of 35-45 years also discusses pregnancy topics but using sophisticated intervention methods including *in vitro fertilization*. Age group of 45-64 years, as in Table 4, discusses topics related to chronic diseases including fibromyalgia, disc, and cholesterol, and it also discusses other topics including addiction to smoking, alcohol, and menopause. HIV also appears to be a popular topic for that group in Google+Health and health Web forums. Finally, people aged older than 65 years also talk more about chronic diseases and heart-related problems including drugs that can help mitigate the pain. We see that most topics are more likely discussed by women because drug review websites and health Web forums are dominated by female users [21].

**Table 5:** Top 10 distinctive terms by age.

Age, years	Google+Health	Drugs	Forums
0-17	N/A	Concerta, Acne, ADHD, Birth, Wash, Lip, Prescribed, Boyfriend, Skin, Scar	Lesbian, Bullying, Buddy, Gay, Mum, Crush, Suicide, Rape, Teen, Dad
18-34	Supplement, Arthritis, Weight, Vitamin, Headache, Hospital, Friend, Food, Love, Skin	Clomid (used to cause ovulation in women), Ovulation, Phentermine, Calorie, Pregnancy, Gym, Pregnant, Baby, BC (birth control), Workout	BC (birth control), Clomid (used to cause ovulation in women), Ovulation, PCOS (polycystic ovary syndrome), TTC (trying to conceive), Miscarried, Fiance, Baby, Pap, Conceive
35-44	Vitamin, Sleep, Food, Parkinson, Friend, Healthcare, Community, Vaccine, Pain, Insomnia	IVF (in vitro fertilization), IUI (intrauterine insemination), Clomid, Ovulation, Marriage, Divorce, Mania, Narcotic, Lithium, Kid	IVF (in vitro fertilization), IUI (intrauterine insemination), BFP (big fat positive), BFN (big fat negative), PG, Stbx (Soon-to-be-ex), Lupron, HCG, Infertility, Fertile
45-64	Syndrome, Death, Chronic, Diet, Anxiety, Hospital, HIV, Infect, Treatment, Flu	Menopause, Fibromyalgia, Oxycontin, Chantix, AA (alcoholic anonymous), RA (rheumatoid arthritis), Disc, Narcotic, Heat, Chronic	Menopause, Grandson, HIV, Disc, Tinnitus, Lesion, Liver, Cholesterol, Enzyme, Colon
65+	N/A	Diovan, Lisinopril, Neuropathic, Urine, Ankle, Cholesterol, Stroke, Arthritis, BP (blood pressure), Cancer	COPD (chronic obstructive pulmonary disease), Valium, PD (panic disorder), Caregiver, Retire, Oxygen, Transplant, Chemo, Cardiologist, Grandchildren

Table 6 summarizes top distinctive disorders by age. In drug review websites, the young age group of 0-17 years talks more about skin disorders and mental disorders, whereas the same age group in health forums websites discusses mainly mental disorders. Age groups of 18-34 years and 35-44 years in drug review websites talk about pregnancy and mental disorder topics. Older age groups in both sources tend to talk about diabetes, heart diseases, and muscles pain.

**Table 6:** Top 5 distinctive disorders by age.

Age, years	Drugs	Forums
0-17	Acne vulgaris, Acne, Attention deficit hyperactivity disorder, Mood swings, Feeling suicidal (finding)	Depressed mood, Incised wound, Mental depression, Fear (finding), Emotional distress
18-34	Endometriosis, site unspecified, Gravidity, Panic attacks, Anxiety attack, Manic	Gastritis, Asthma, Panic, Anxiety disorders, Observation of attack
35-44	Endometriosis, site unspecified, Manic, Manic mood, Addictive behavior, Chronic pain	Autistic disorder; Disability; Lupus erythematosus, systemic; Attention deficit hyperactivity disorder; Pressure (finding)
45-64	Hot flushes, Chronic pain, Fibromyalgia, Night sweats, Nerve pain	Codependency; Gastritis; Fibromyalgia; Lupus vulgaris; Lupus erythematosus, systemic
65+	Muscle cramps in leg, Dry cough, Lassitude, Diabetic, Blood pressure finding	Atrial fibrillation, Diabetic, Panic attacks, Cerebrovascular accident, Gastroesophageal reflux disease

In Table 7, we summarize all age groups’ top drugs. For the younger group of 0-17 years in drug review websites, top drugs discussed are the ones related to ADHD. Age group of 18-34 years in drug review websites discusses pregnancy-related drugs, whereas for age group of 35-44 years, the top drugs are related to MS disorder. This group of 35-44 years in health Web forums tends to share information about ADHD drugs. Older age users (65+ years) discuss drugs related to heart problems, blood pressure, diabetes, and cholesterol.

**Table 7:** Top 5 distinctive drugs by age.

Age, years	Drugs^a^	Forums^a^
0-17	Accutane, Concerta^1^, Vyvanse^1^, Strattera^1^, Implanon^3^	Commit Lozenge, Relate—vinyl resin, Vent, Zoloft^4^, Topamax
18-34	Clomid^3^, Phentermine, Seasonique^3^, Lupron^2^, Yaz^3^	Human papilloma virus vaccine, Topamax, Diamox, Adderall^1^, Antibiotics
35-44	Clomid^3^, Rebif^5^, Avonex^5^, Tysabri^5^, Lortab	Concerta^1^, Melatonin, Diamox, Plaquenil, Adderall^1^
45-64	Tamoxifen^2^, Avonex^5^, Oxycontin, Savella, Soma	Smoke, Hydrocortisone, Cymbalta^4^, Lyrica^7^, Alcohols
65+	Plavix^6^, Diovan^6^, Actos^7^, Hydroxymethylglutaryl-CoA reductase inhibitors, Lipitor^6^	Metformin^7^, Carbohydrates, Oxygen, Sugars, Xanax^4^

^a^Some of the drugs are coded to match the corresponding disorders they treat:^1^ADHD,^2^Cancer,^3^pregnancy,^4^depression,^5^MS,^6^BP, heart problem and cholesterol,^7^Diabetes.

Sentiment and emotion were evaluated for all sources. Because the results look similar among age groups, we summarize the results in Tables C.3 and C.4 of Multimedia Appendix 1. One key finding from the emotion results is that older people in Google+Health and drug review websites express less anger, whereas younger people in drug review websites express more anger.

### Ethnicity

Only TwitterHealth and Google+Health have a large enough number of users whose ethnicity we can estimate (see Table A.2 in Multimedia Appendix 1), and hence, we only report finding for these outlets. In Table 8, we summarize top disorders for each ethnicity except black owing to the small number of users. As a key finding of top disorders, fibromyalgia is one of the top disorders that white and Hispanic users discuss in TwitterHealth, heart and kidney diseases are discussed more by Asian users, and headache and sleeplessness are 2 of the top disorders discussed by Hispanic users. The other ethnicity-based results exhibit less variance among the ethnicity groups, and hence, we report in Tables C.5, C.6, C.7, and C.8 of Multimedia Appendix 1.

**Table 8:** Top 5 distinctive disorders by ethnicity.

Ethnicity	TwitterHealth	Google+Health
White	Fibromyalgia, Presenile dementia, Leukemia, Migraine disorders, Mental disorders	Binge eating disorder, Diabetic neuropathies, Marijuana abuse, Neuropathy, Crohn disease
Asian	Heart diseases, Food poisoning, Obesity, Herpes NOS, Stress	Kidney diseases, Myopia, Fatigue, Hemorrhage, Hypersensitivity
Hispanic	Headache, Fibromyalgia, Sleeplessness, Mental depression, Insomnia adverse event	Herpes zoster disease, Diarrhea, Suicide, Lupus vulgaris, Osteoporosis

### Location

Table 9 summarizes the top disorder results for all sources. Focusing on drugs and forums, which have been shown to have more useful information regarding one’s health [13], our key finding is that users in the Northeast talk more about traditional physical disorders including diabetes and heart conditions, users in the Midwest discuss about weight loss, users in the South about fibromyalgia, and users in the West discuss mental disorders and addictive behaviors.

The other location-based results including sentiment, emotions, top distinctive terms, and top distinctive drugs exhibit less variance among the location groups, and hence, we report them in Tables C.9, C.10, C.11, and C.12 of Multimedia Appendix 1, as the variations across locations are not significant.

### Writing Level

Table 10 summarizes the emotion results for all sources. For shortness, only 3 emotions are listed here: anger, trust, and anticipation, as the other 3 (fear, disgust, and surprise), are complementary to these, respectively. We see that users with lower writing level express more anger, with the exception of drug review websites, whereas people with higher writing level express less anger. Due to low variance among writing levels, the other results for writing level including sentiment, top distinctive terms, top distinctive disorders, and top distinctive drugs can be found in Tables C.13, C.14, C.15, and C.16 of Multimedia Appendix 1, respectively.

**Table 9:** Top 5 distinctive disorders by location.

Location	TwitterHealth	Google+Health	Drugs	Forums
Northeast	N/A	Inflammatory bowel diseases, Crohn disease, Occupant of van injured in transport accident, Kidney diseases, Prostate carcinoma	Asleep, Seizures, Patient outcome—died, Memory observations, Fatigue	Diabetes, Atrial fibrillation, Gastroesophageal reflux disease, ACHE, Lupus vulgaris
Midwest	Migraine disorders, Primary malignant neoplasm	Confusion, Marijuana abuse, Van der Woude syndrome, Injury wounds, Cataract	Hemorrhage, Body weight decreased, Hot flushes, Weight loss adverse event, Xerostomia	Asthma, Migraine disorders, Pressure (finding), Autistic disorder, Cerebrovascular accident
South	N/A	Diabetic neuropathies, Binge eating disorder, Neuropathy, Alzheimer's disease pathway KEGG, Diarrhea	Fibromyalgia, Drowsiness, Edema, Pruritus, Manic	Codependency, Shot (injury)
West	Presenile dementia, Heart diseases, Mental suffering, Herpes NOS, Obesity	Sexually transmitted diseases, Bipolar disorder, Myocardial infarction, Vitality, Acquired immunodeficiency syndrome	Post-traumatic stress disorder, Sleeplessness, Anxiety attack, Addictive behavior, Suicidal	Marijuana abuse, Addictive behavior, codependency, Autistic disorder, Lupus erythematosus, systemic

**Table 10:** Emotion for each demographic grouped by source.

Writing level	TwitterHealth			Google+Health			Drugs			Forums		
	Anger (%)	Trust (%)	Anticipation (%)	Anger (%)	Trust (%)	Anticipation (%)	Anger (%)	Trust (%)	Anticipation (%)	Anger (%)	Trust (%)	Anticipation (%)
0-5	N/A	N/A	N/A	38.2^a^	68.5	71.2^a^	31.6^a^	66.8^a^	72.9^a^	34.3^a^	78.1^a^	71.5^a^
6-9	41.0^a^	44.3^a^	75.3^a^	34.6^a^	67.9	75.8^a^	31.4^a^	67.7^a^	73.2^a^	31.7^a^	77.8^a^	72.6^a^
10-16	34.1^a^	55.2^a^	81.9^a^	31.0^a^	66.6	79.1^a^	29.9^a^	73.1^a^	72.9^a^	27.4^a^	77.2^a^	75.4^a^

^a^Represents the values with high significance (*P*≤.05) compared with the union of the other age groups.

## Discussion

### Notable Results

Our results provide valuable information that can help reach the right demographic group for each health condition. For example, to reach young users (aged 0-17 years) with ADHD, one should go to drug review websites. This finding can be a result of the increased percentage of children with ADHD recently (9%), compared with 2000 when it was 7% [44]. Similarly, to reach users of age group 18-34 with sleep disorder, one should go to Google+. We also found that the age group of 35-44 years discusses drugs associated with MS disorder, which agrees with the average age of clinical onset of MS, which is 30-33 years, and the average age of diagnosis, which is 37 years [45]. Because older age groups as our results show tend to discuss chronic diseases such as diabetes, heart problems, and cholesterol, health professionals and educators can target these groups in drug review websites and health Web forums to increase national awareness and decrease disease-related deaths.

Furthermore, a surprising finding is that, despite the fact that women suffer from back pain more than men [46], our results show that men discuss back pain more than women. Because 76% of all adults who have HIV are men [47], our results support this fact where HIV is one of the top discussed topics in TwitterHealth.

Our results also found that users in Western states discuss mental disorders and addictive behaviors including alcohol and marijuana as Table C.11 of Multimedia Appendix 1 shows. This finding is associated with the fact that 5 of top 10 states with high marijuana use are in the West area [48]. Midwest users discuss weight loss more than the other regions according to our results, which can be related to the fact that the Midwest is the second (slightly trailing the South) highest region in terms of obesity, with more than 25% of the adults being obese (body max index of 30+) [49].

### Applications

There are several ways to leverage our results. Our findings can help health care providers and public health officials create targeted and effective educational campaigns, guide advertisers for different topics discussed by different demographic groups, help funding agencies allocate their research funds to have a larger impact on the society’s top health issues, and help understand health disparities in Web-based health social media.

For instance, to reach pregnant or trying-to-get-pregnant women, advertisers should go to health Web forums and drug review websites instead of Google+ for advertising related products. This finding is supported by the fact that drug review websites and health Web forums are dominated by female users [21]. Also, this finding may indicate that there is a need for more definitive and authoritative sources of such information.

Our results can also help understand health disparities in Web-based health social media. Users with higher writing level are less angry when discussing health-related issues, which may be linked to the fact that people with lower level of education receive lower quality of health care [50] and have higher mortality rate [14].

These demographics-specific findings can be used in targeted educational campaigns, which are recently becoming the focus of several research efforts. As an example, Whittaker [51] shows how a smoking cessation intervention using mobile phones for young adults can be effective by sending general health videos messages and setting a quit date. Furthermore, Opel et al [52] show how social marketing can be used to increase immunization rates, where they explained how social marketing techniques can capture attention and motivate the targeted population to change. Patel et al [53] performed a systemic review to evaluate the effect of applications of contemporary social media on clinical outcomes in chronic disease. The study shows that providing social, emotional, and experiential support in current social media can help improve the patient care. Valle et al [54] evaluated a Facebook-based intervention that aims to increase the physical activity of young adult cancer survivors, which shows a potential for increasing the physical activity compared with Facebook-based self-help. A review of health interventions in Web-based social networks is presented in the study by Maher et al [55] where it is shown that several studies included in the systematic review reported significant improvement in health behavior or outcomes.

### Limitations

For the general social networks, Google+ and Twitter, we used 276 health keywords and phrases as we described in the Methods section to filter the posts. These keywords and phrases miss some consumer phrases or abbreviations, such as ivf (in vitro fertilization) and iui (Intrauterine insemination). Unfortunately, we must select a relatively small set of keywords, given the rate constraints of the APIs of the social media.

Owing to the fact that ethnicity was estimated using a classifier [21], we were not able to confidently compute the ethnicity of enough users to have reliable results for several cases. For that, we omit results for black users. Furthermore, we do not report ethnicity results for drug review websites and health Web forums because these sources do not provide users’ last names. Another limitation is self-selection bias because all demographic attributes (explicitly reported or classified) are reported by users. For instance, a user may choose to report age or last name (which is used to classify ethnicity). For example, people who trust the opinion of other users or experts participate more in social networks, whereas people who have less trust might not share their private experiences.

For extracting medical concepts, we do not handle all abbreviations. We handle some cases through manual rules, for example, Metamap would map “I” to iodine. Also, MetaMap is not perfect for annotating social media posts; thus, we removed annotations that look incorrect as the previous example. Moreover, when computing the top distinctive terms, we do not handle variations of terms, that is, “iui” and “Intrauterine insemination” are considered different terms. We do a manual postprocessing to address this issue for the top results. In measuring the sentiment of posts, the sentiment lexicon “SentiWordNet” was not built specifically for social or medical text. For example, some words such as “omg” or “lol” are not mapped to any word in the lexicon; thus, not all terms in the posts are assigned a sentiment.

### Conclusion

We analyzed the content of Web-based health social media based on users' demographics. Three different types of Web-based health social media were considered: social networks, drug review websites, and health Web forums. For each demographic attribute—gender, age, ethnicity, location, and writing level—we evaluated sentiment and emotion, and we extracted top distinctive terms and medical concepts, specifically disorders and drugs. Our results are both expected and surprising and show several key findings for each demographic attribute. For example, the dominant topic for female users in drug review websites and health Web forums is pregnancy, whereas for male users, it is cardiac problems, HIV, and back pain. Attention-deficit hyperactivity disorder and depression-related drugs are the main topics discussed by younger users (0-17 years), MS drugs are discussed more by users of age 35-44 years, and alcohol and smoking are mainly discussed by middle-aged users (45-64 years). Users from the Northeast United States talk about physical disorders, whereas users from the West United States talk about mental disorders and addictive behaviors. Finally, users with higher writing level express less anger in their posts. These key findings can help experts reach the right users in many ways, including creating targeted and effective educational campaigns by health care providers, advertising related products, allocating funds for the right research by funding agencies, and understanding health disparities in Web-based health social media.

## Appendix

Multimedia Appendix 1 Web-Based Social Network Summary, Health-Related Keywords, Results, and Statistical Significance Tests.

## Abbreviations

AA: alcoholic anonymous
API: Application Program Interface
ADHD: attention-deficit hyperactivity disorder
BC: birth control
BP: blood pressure
BFN: big fat negative
BFP: big fat positive
COPD: chronic obstructive pulmonary disease
IUI: intrauterine insemination
IVF: in vitro fertilization
MS: multiple sclerosis
NLP: Natural Language Processing
PCOS: polycystic ovary syndrome
PD: panic disorder
RA: rheumatoid arthritis
Stbx: Soon-to-be-ex
TTC: trying to conceive
UMLS: Unified Medical Language System
