# Supplementary material for: Demographic-Based Content Analysis of Web-Based Health-Related Social Media
Source: J Med Internet Res. 2016 Jun 13;18(6):e148. doi: 10.2196/jmir.5327 (PMC4923586; doi:10.2196/jmir.5327)
Supplement: Multimedia Appendix 1 [file jmir_v18i6e148_app1.pdf]

Appendix A

A.1 Web-Based Social Network Summary

Table A.1 summarizes the used sources in our work, including the web address, and the start and end dates for collected posts. For TwitterHealth, we use a sample of 10%.

Table A.1 The start and end dates for sources represent the time of first post and last post. Not Applicable (N/A) is used for sources that do not log each post's creation date.

Dataset	URL	Start	End
TwitterHealth [1]	www.twitter.com	May 1, 2013	Nov. 15, 2013
Google+Health [2]	plus.google.com	Aug. 24, 2009	Jan. 05, 2014
Drugs.com [3]	www.drugs.com	Feb. 16, 2007	Jan. 26, 2014
DailyStrength / Treatments [4]	www.dailystrength.org/treatments	N/A	N/A
WebMD / Drugs [5]	www.webmd.com/drugs	Sep. 17, 2007	Nov. 27, 2013
Drugs.com / Answers [6]	www.drugs.com/answers	Mar. 25, 2004	Feb. 02, 2014
WebMD [7]	www.webmd.com	Dec. 31, 1999	Feb. 07, 2014
DailyStrength/Forums [8]	www.dailystrength.org/support-groups	Jun. 21, 2006	Jan. 25, 2014

A.2 Web-Based Social Network Data Summary

Table A.2 summarizes the total number of users and posts for all reported demographics.

Table A.2 List no. of users and post for each demographic in the all sources

	**TwitterHealth**		**Google+Health**		**Drugs**		**Forums**	
	No. of users	No. of posts	No. of users	No. of posts	No. of users	No. of posts	No. of users	No. of posts
**Gender**								
Male	257,562	1,124,698	37,172	61,479	63,323	97,728	36,916	514,788
Female	276,924	1,330,214	20,333	32,082	232,980	506,473	134,066	2,468,351
**Age**	N/A	N/A						
0-17			N/A	N/A	2,881	4,510	1,352	9,007
18-34			389	552	85,184	187,203	52,219	725,387
35-44			160	308	61,191	137,644	33,859	716,081
45-64			139	499	100,814	200,034	37,988	966,781
65+			N/A	N/A	23,597	36,171	5,785	187,485
**Ethnicity**					N/A	N/A	N/A	N/A
White	89,522	714,301	3,214	17,230				
Black	401	3,579	12	72				
Asian	3,973	46,436	264	2,825				
Hispanic	28,774	217,029	730	3,389				
**Location**								
Northeast	165,531	394,879	2,598	4,393	114,876	66,658	402,781	409,602
Midwest	174,620	386,688	2,393	4,209	147,624	87,303	441,079	441,032
South	313,350	741,781	4,863	9,269	211,045	127,071	677,662	680,903
West	181,400	437,731	4,690	7,805	138,587	680,903	470,595	470,538
**Writing Level**								
0-5	47,955	998,828	1,302	2,657	44,284	203,941	76,513	3,333,111
6-9	64,876	1,429,745	11,224	27,672	94,551	306,972	181,367	3,949,807
10-16	14,133	348,301	7,649	24,918	181,367	6,873	7,917	28,911

Appendix B

The list of used keywords to filter health-related posts from Twitter and Google+:

**Table B.1** Used keywords to filter health-related posts from Twitter and Google+				
		**Drugs**		
Abilify	Clonidine	Hydrochlorothiazide	Nasonex	Sulfamethoxazole
Actonel	Combivent	Hydrocodone	Nexium	Synthroid
Actos	Concerta	Ibuprofen	Niaspan	Toprol
Advair	Crestor	Isosorbide	Nuvaring	Tramadol
Albuterol	Cyclobenzaprin	Januvia	Omeprazole	Trazodone
Alendronate	Cymbalta	Klor-Con	Oxycodone	Triamterene
Allopurinol	Detrol	Lantus	Oxycontin	Tricor
Alprazolam	Diazepam	Levaquin	Pantoprazole	TriNessa
Ambien	Digoxin	Levothyroxine	Paroxetine	Ventolin
Amlodipine	Diltiazem	Levoxyl	Penicillin	Verapamil
Amoxicillin	Diovan	Lexapro	Plavix	Viagra
Amphetamine	Doxycycline	Lipitor	Potassium	Vitamin
Aricept	Effexor	Lisinopril	Pravastatin	Vytorin
Atenolol	Enalapril	Loestrin	Premarin	Vyvanse
Azithromycin	Famotidine	Lorazepam	Proair	Warfarin
Benazepril	Fexofenadine	Lovastatin	Promethazine	Xalatan
Benicar	Flomax	Lovaza	Propoxyphen	Zetia
Carisoprodol	Flovent	Lyrica	Proventil	Zolpidem
Carvedilol	Fluconazole	Meloxicam	Ranitidine	Zyprexa
Cefdinir	Fluoxetine	Metformin	Seroquel	Amitriptyline
Celebrex	Fluticasone	Methylprednisolone	Sertraline	Cheratussin
Cephalexin	Folic	Metoprolol	Simvastatin	Ocella
Cialis	Furosemide	Mupirocin	Singulair	Prednisone
Ciprofloxacin	Gabapentin	Namenda	Spiriva	
Citalopram	Glyburide	Naproxen	Suboxone	
Clonazepam				
		**Hashtags**		
#BCSM	#HCSM	#hcsmeu	#HITsm	#mhsm
#doctors20	#hcsmca	#hcsmin	#Ideagoras	#RareDisease
#eldercarechat				
		**Disorders**		
AIDS	Constipation	Heart disease	Liver disease	Otitis
Alzheimer	COPD	Hemochromatosis	Lupus	Overweight
Anxiety disorders	Crohn's disease	Hepatitis	Lyme disease	Parkinson's
Arthritis	Cystic fibrosis	Herpes	Lymphoma	Pelvic inflammatory
Asthma	Dementia	High cholesterol	Meningitis	disease
Astigmatism	Depression	HIV	Meningococcal	Pertussis
Autoimmune	Diabetes	Hodgkin's disease	Menopause	Prostate disorder
Bipolar	Eczema	HPV	Mental illness	Raynaud's
Cancer	Endometriosis	Hypertension	Migraine	Phenomenon
Candidiasis	Fibroids	Impotence	Multiple sclerosis	SARS
Cataracts	Fibromyalgia	Insomnia	Muscular dystrophy	Sexually transmitted
Celiac	Flu	Irritable bowel	Myopia	disease
Chicken pox	Food poisoning	syndrome	Narcolepsy	Sleep disorder
Chlamydia	Gallstones	Jaundice	Non-Hodgkin's	Stroke
Chronic fatigue	Gonorrhea	Kidney disease	lymphoma	Thrush
syndrome	Grave's disease	Lactose intolerance	Obesity	Thyroid
Cold sore	Hay fever	Leukemia	Osteoporosis	Whooping cough
Common cold	Headache			

Pharmaceuticals				
Johnson & Johnson<br>Pfizer<br>Roche	GlaxoSmithKline<br>Novartis<br>Sanofi	AstraZeneca<br>Abbott	Merck<br>Bayer	Eli Lilly<br>Bristol-Myers

Insurance				
healthcare<br>health insurance<br>medicare<br>medicaid<br>AARP<br>Aetna<br>Aflac<br>American Family Insurance<br>American Fidelity Assurance<br>American National Insurance	Company<br>Amerigroup<br>Anthem Blue Cross<br>Assurant<br>Bankers Life and Casualty<br>Blue Cross and Blue Shield<br>Centene<br>Cigna<br>Conseco	Coventry Health<br>EmblemHealth<br>Fortis<br>Golden Rule Insurance<br>Group Health Cooperative<br>HealthNet<br>HealthMarkets<br>HealthSpring<br>Highmark Insurance	Humana<br>Independence Blue Cross<br>Kaiser Permanente<br>Kaleida Health<br>LifeWise Health Plan of Oregon<br>Medical Mutual of Ohio<br>Molina Healthcare<br>Mutual of Omaha<br>Premera Blue Cross	Principal Financial Group<br>The Regence Group<br>Tricare<br>Shelter Insurance<br>Thrivent Financial<br>UnitedHealth<br>Unitrin<br>Universal American Corporation<br>WellCare Health<br>WellPoint

The frequency of each keyword in Google+health and TwitterHealth:

Table B.2 Keywords frequencies for Twitter and Google+

Keyword	Google+Health	TwitterHealth	Keyword	Google+Health	TwitterHealth
Abilify	29	230	Anxiety disorders	369	1565
Actonel	3	9	Arthritis	3554	47516
Actos	518	3763	Asthma	3007	114037
Advair	7	107	Astigmatism	484	2218
Albuterol	103	866	Autoimmune	1430	4318
Alendronate	5	15	Bipolar	1817	318168
Allopurinol	27	45	Cancer	15569	1395509
Alprazolam	60	247	Candidiasis	107	177
Ambien	251	6965	Cataracts	1021	4824
Amlodipine	32	87	Celiac	819	8493
Amoxicillin	153	1513	Chicken pox	335	10955
Amphetamine	316	2333	Chlamydia	291	23345
Aricept	10	28	Chronic fatigue syndrome	188	593
Atenolol	28	85	Cold sore	355	11306
Azithromycin	98	494	Common cold	912	11239
Benazepril	7	14	Constipation	1671	15330
Benicar	12	17	COPD	173	616
Carisoprodol	8	22	Crohn's disease	28	21
Carvedilol	10	20	Cystic fibrosis	287	4096
Cefdinir	6	11	Dementia	2089	62011
Celebrex	16	477	Depression	7093	681145
Cephalexin	21	200	Diabetes	7550	171662
Cialis	1472	21542	Eczema	1063	16257
Ciprofloxacin	52	154	Endometriosis	429	3645
Citalopram	69	720	Fibroids	58	1430
Clonazepam	57	252	Fibromyalgia	1403	7033
Clonidine	22	76	Flu	10169	364347

Combivent	4	11	Food poisoning	642	39071
Concerta	22	577	Gallstones	319	1466
Crestor	15	40	Gonorrhea	393	8940
Cyclobenzaprin	10	7	Grave's disease	1	6
Cymbalta	50	761	Hay fever	449	19819
Detrol	3	5	Headache	7494	1438033
Diazepam	108	966	Heart disease	3891	54353
Digoxin	51	157	Hemochromatosis	36	129
Diltiazem	11	39	Hepatitis	1154	14451
Diovan	5	227	Herpes	1149	105449
Doxycycline	91	388	High cholesterol	785	7043
Effexor	20	213	HIV	1891	16142
Enalapril	9	15	Hodgkin's disease	1	1
Famotidine	8	116	HPV	147	1732
Fexofenadine	10	49	Hypertension	1615	7706
Flomax	7	229	Impotence	717	6447
Flovent	8	19	Insomnia	2730	237249
Fluconazole	28	255	Irritable bowel syndrome	267	850
Fluoxetine	81	340	Jaundice	504	2767
Fluticasone	53	91	Kidney disease	1274	3917
Folic	704	2032	Lactose intolerance	248	2352
Furosemide	33	222	Leukemia	1229	21233
Gabapentin	87	334	Liver disease	642	3452
Glyburide	10	37	Lupus	984	13061
Hydrochlorothiazide	26	296	Lyme disease	43	689
Hydrocodone	318	2788	Lymphoma	719	5534
Ibuprofen	905	32341	Meningitis	1398	9182
Isosorbide	4	8	Meningococcal	122	607
Januvia	18	6	Menopause	1425	21166
Klor-Con	1	1	Mental illness	2088	60505
Lantus	12	152	Migraine	2867	129988
Levaquin	11	263	Multiple sclerosis	886	3604
Levothyroxine	38	125	Muscular dystrophy	255	1579
Levoxyl	6	11	Myopia	452	4286
Lexapro	30	1189	Narcolepsy	387	4357
Lipitor	110	214	Non-Hodgkin's lymphoma	6	5
Lisinopril	34	85	Obesity	4791	104106
Loestrin	5	49	Osteoporosis	1346	4831
Lorazepam	51	367	Otitis	127	456
Lovastatin	25	25	Overweight	3368	99249
Lovaza	1	11	Parkinson's	24	29
Lyrica	70	782	Pelvic inflammatory	71	356

			disease		
Meloxicam	10	71	Pertussis	255	1237
Metformin	384	834	Prostate disorder	4	1
Methylprednisolone	61	45	Raynaud's Phenomenon	1	1
Metoprolol	36	56	SARS	105	2045
Mupirocin	3	32	Sexually transmitted disease	416	1861
Namenda	8	18	Sleep disorder	570	3131
Naproxen	143	1000	Stroke	4861	276947
Nasonex	7	158	Thrush	338	4415
Nexium	33	612	Thyroid	2496	15747
Niaspan	2	2	Whooping cough	446	3563
Nuvaring	36	368	Johnson & Johnson	5	2
Omeprazole	79	2129	Pfizer	172	706
Oxycodone	329	1762	Roche	394	2750
Oxycontin	147	856	GlaxoSmithKline	115	126
Pantoprazole	16	33	Novartis	139	122
Paroxetine	44	148	Sanofi	79	649
Penicillin	614	6208	AstraZeneca	112	30
Plavix	20	250	Abbott	289	10132
Potassium	1953	23914	Merck	110	391
Pravastatin	24	10	Bayer	152	1982
Premarin	8	190	Eli Lilly	1	12
Proair	6	5	Bristol-Myers	20	3
Promethazine	35	4667	healthcare	6711	248356
Propoxyphen	4	4	health insurance	1971	95957
Proventil	2	13	medicare	892	6669
Ranitidine	20	100	medicaid	668	4787
Seroquel	47	481	AARP	352	2719
Sertraline	50	202	Aetna	95	336
Simvastatin	66	169	Aflac	65	976
Singulair	7	247	American Family Insurance	2	31
Spiriva	2	20	American Fidelity Assurance	1	1
Suboxone	109	352	American National Insurance	1	1
Sulfamethoxazole	28	30	Amerigroup	121	86
Synthroid	48	432	Anthem Blue Cross	3	17
Toprol	74	340	Assurant	21	57
Tramadol	154	3383	Bankers Life and Casualty	1	3

Trazodone	17	299	Blue Cross and Blue Shield	1	17
Triamterene	8	11	Centene	11	17
Tricor	11	36	Cigna	79	299
TriNessa	1	3	Conseco	5	118
Ventolin	61	358	Coventry Health	1	4
Verapamil	24	66	EmblemHealth	2	3
Viagra	480	42373	Fortis	232	459
Vitamin	5920	122013	Golden Rule Insurance	1	1
Vytorin	3	9	Group Health Cooperative	1	2
Vyvanse	27	4113	HealthNet	18	88
Warfarin	229	899	HealthMarkets	6	5
Xalatan	1	1	HealthSpring	5	2
Zetia	4	26	Highmark Insurance	1	2
Zolpidem	83	193	Humana	272	2093
Zyprexa	23	238	Independence Blue Cross	1	5
Amitriptyline	49	326	Kaiser Permanente	3	66
Cheratussin	2	19	Kaleida Health	1	8
Ocella	3	84	LifeWise Health Plan of Oregon	1	1
Prednisone	314	1773	Medical Mutual of Ohio	1	1
#BCSM	104	4257	Molina Healthcare	1	2
#doctors20	48	757	Mutual of Omaha	2	8
#eldercarechat	15	989	Premera Blue Cross	1	2
#HCSM	2294	15120	Principal Financial Group	1	2
#hcsmca	64	1976	The Regence Group	1	1
#hcsmeu	188	1593	Tricare	63	347
#hcsmin	10	635	Shelter Insurance	1	7
#HITsm	185	1828	Thrivent Financial	1	1
#Ideagoras	5	11	UnitedHealth	55	36
#mhsm	274	5509	Unitrin	1	2
#RareDisease	98	1401	Universal American Corporation	1	1
AIDS	1983	166512	WellCare Health	1	1

Alzheimer	740	8429	WellPoint	36	73

Appendix C

In this appendix, we present the results for all demographics attributes that are not presented in the manuscript. The results include sentiment and emotions, top distinctive keywords, top distinctive disorders and drugs.

C.1 Gender

Table C.1 shows the sentiment by gender for each source

Table C.1 Sentiment by gender. O indicates 'Objective', P indicates 'Positive', and N indicates 'Negative'. We put a "*" next to the values with high significance ($p \leq 0.05$) compared to the union of the other age groups.

	**TwitterHealth**			**Google+Health**			**Drugs**			**Forums**		
Gender	O	P	N	O	P	N	O	P	N	O	P	N
Male	86.8%*	6.3%	6.5%*	83.7%*	7.0%	6.4%	85.3%*	6.5%*	7.3%*	83.5%*	6.2%*	5.8%*
Female	86.1%*	6.6%	6.7%*	83.2%*	6.1%	6.6%	85.8%*	6.2%*	7.2%*	84.7%*	6.4%*	5.8%*

Table C.2 shows the emotion by gender for each source

Table C.2 Emotion by gender. We put a "*" next to the values with high significance ($p \leq 0.05$) compared to the union of the other age groups.

	**TwitterHealth**			**Google+Health**			**Drugs**			**Forums**		
Gender	Anger	Trust	Anticipation	Anger	Trust	Anticipation	Anger	Trust	Anticipation	Anger	Trust	Anticipation
Male	40.1%	46.1%*	74.5%*	32.9%	65.5%*	76.3%*	22.0%*	52.7%*	50.2%*	21.4%*	59.1%*	51.6%*
Female	42.0%	40.8%*	72.7%*	34.1%	66.2%*	75.6%*	24.2%*	55.1%*	54.5%*	22.3%*	60.7%*	54.4%*

C.2 Age

Table C.3 shows the sentiment by age for each source

Table C.3 Sentiment by age. O indicates 'Objective', P indicates 'Positive', and N indicates 'Negative'. We put a "*" next to the values with high significance ($p \leq 0.05$) compared to the union of the other age groups.

	**Google+Health**			**Drugs**			**Forums**		
Age group	O	P	N	O	P	N	O	P	N
0-17	N/A	N/A	N/A	84.6%*	6.6%*	7.1%*	86.0%*	6.8%	6.1%*
18-34	82.7%*	6.4%	6.6%*	85.4%*	6.4%*	7.1%*	86.0%*	6.7%*	6.1%*
35-44	84.2%	6.0%	6.9%	85.2%*	6.4%*	7.4%*	85.8%*	6.8%*	6.0%*
45-64	85.6%*	5.7%*	6.8%*	84.9%	6.4%	7.6%*	85.8%*	6.9%*	6.0%
65+	N/A	N/A	N/A	84.5%*	6.3%*	7.9%*	85.9%*	6.8%*	6.1%*

Table C.4 shows the sentiment by gender for each source

Table C.4 Emotion by age. We put a “*” next to the values with high significance (p≤0.05) compared to the union of the other age groups.

	**Google+Health**			**Drugs**			**Forums**		
Age group	Anger	Trust	Anticipation	Anger	Trust	Anticipation	Anger	Trust	Anticipation
0-17	N/A	N/A	N/A	34.8%*	67.2%*	72.1%*	38.5%*	70.6%*	72.3%
18-34	35.5%	62.7%	73.6%	32.7%*	66.1%*	73.4%*	36.9%*	72.8%*	72.9%*
35-44	38.8%	70.3%	73.3%	30.9%*	66.3%*	72.5%*	36.2%*	75.7%	72.2%
45-64	29.8%	64.0%	75.6%*	29.8%*	66.5%*	72.6%*	35.1%*	77.5%*	72.2%*
65+	N/A	N/A	N/A	27.9%*	67.0%*	72.3%	35.1%*	77.0%	71.8%*

C.3 Ethnicity

Table C.5 shows the sentiment by ethnicity for each source

Table C.5 Sentiment by ethnicity. O indicates ‘Objective’, P indicates ‘Positive’, and N indicates ‘Negative’. We put a “*” next to the values with high significance (p≤0.05) compared to the union of the other age groups.

	**TwitterHealth**			**Google+Health**		
Ethnicity	O	P	N	O	P	N
White	86.5%*	6.4%*	6.6%*	84.5%*	6.0%*	6.3%*
Black	86.4%*	6.6%*	6.6%	80.5%	5.6%	7.0%
Asian	86.0%*	6.7%*	6.7%*	82.6%	5.9%	6.7%*
Hispanic	85.8%*	6.5%*	6.8%*	82.4%*	5.9%*	6.6%*

Table C.6 shows the emotion by ethnicity for each source

Table C.6 Emotion for each demographic grouped by source. We put a “*” next to the values with high significance (p≤0.05) compared to the union of the other age groups.

	**TwitterHealth**			**Google+Health**		
Ethnicity	Anger	Trust	Anticipation	Anger	Trust	Anticipation
White	41.0%*	43.6%*	73.5%*	33.9%	67.2%	75.6%*
Black	43.5%*	37.3%*	70.7%	31.5%	72.2%	76.4%
Asian	39.3%	44.5%*	73.3%*	31.8%	62.2%	77.2%*
Hispanic	43.3%*	39.6%*	72.4%*	34.7%	63.0%	75.9%

Table C.7 shows the top 10 distinctive keywords by ethnicity for each source

**Table C.7** Top 10 distinctive terms for ethnicity		
**Ethnicity**	**TwitterHealth**	**Google+Health**
White	tumor<br>pancreatitis<br>hay fever<br>clinic<br>liver<br>ovarian<br>syndrome<br>doctor<br>menopause<br>leukemia	psoriasis<br>mri<br>slim<br>diazepam<br>yeast<br>cigna<br>malignant<br>anxious<br>infertility<br>seroquel
Black	N/A	N/A
Asian	chicken pox<br>remedy<br>kidney<br>constipation<br>epidemiology<br>cholesterol<br>hepatitis<br>gay<br>clinic<br>treatment	N/A
Hispanic	rn<br>cry<br>fibromyalgia<br>oxygen<br>iq<br>irritable<br>throat<br>mouth<br>headache<br>chlamydia	conceive<br>itchy<br>germ<br>gym<br>kaiser permanente<br>hodgkin<br>urine<br>drain<br>fever<br>sweat

Table C.8 shows the top 5 distinctive drugs by ethnicity for each source

**Table C.8** Top 5 distinctive drugs by ethnicity		
**Ethnicity**	**TwitterHealth**	**Google+Health**
White	Ambien<br>Cialis<br>human papilloma virus vaccine	Byetta<br>Grit (substance)<br>Aricept<br>Starch<br>Pradaxa
Asian	human papilloma virus vaccine<br>trivalent influenza vaccine<br>Vitamin C Vitamins<br>Cialis<br>Vitamins	Intermezzo<br>Yaz<br>Melatonin<br>Epinephrine<br>zolpidem

Hispanic	Cialis<br>Viagra<br>Vitamins	Purified Protein Derivative of Tuberculin<br>Diphosphonates<br>Opioids<br>Oxycontin<br>Hormones

C.4 Location

Table C.9 shows the sentiment by location for each source

Table C.9 Sentiment by location. O indicates ‘Objective’, P indicates ‘Positive’, and N indicates ‘Negative’. We put a “*” next to the values with high significance (p≤0.05) compared to the union of the other age groups.

	TwitterHealth			Google+Health			Drugs			Forums		
Area	O	P	N	O	P	N	O	P	N	O	P	N
Northeast	86.8%*	6.4%	6.4%*	85.5%*	6.2%*	6.1%	84.5%*	6.6%	7.1%*	85.9%*	6.9%*	6.0%*
Midwest	86.6%*	6.4%*	6.5%*	85.5%*	6.1%*	6.0%*	84.5%	6.6%	7.2%*	85.9%*	6.8%*	5.9%*
South	86.8%*	6.4%*	6.3%	84.5%*	6.1%*	6.3%	84.5%*	6.7%*	7.2%*	86.0%*	6.7%*	6.0%*
West	86.6%	6.4%*	6.5%*	83.9%*	6.2%*	6.5%*	84.3%*	6.6%*	7.2%*	86.1%*	6.8%*	6.0%*

Table C.10 shows the emotion by location for each source

Table C.10 Emotion for each demographic grouped by source. We put a “*” next to the values with low significance (p>0.05) compared to the union of the other regions. We put a “*” next to the values with high significance (p≤0.05) compared to the union of the other age groups.

	TwitterHealth			Google+Health			Drugs			Forums		
Area	Anger	Trust	Anticipation	Anger	Trust	Anticipation	Anger	Trust	Anticipation	Anger	Trust	Anticipation
Northeast	40.8%	47.2%	75.0%	41.6%	45.0%	74.5%	42.1%*	42.9%	74.1%	40.1%*	48.4%*	75.4%*
Midwest	34.1%*	68.9%	73.1%*	33.6%*	69.2%*	77.0%*	35.5%	69.3%*	76.2%*	33.2%	68.2%	76.4%
South	33.0%*	68.0%*	73.5%	32.6%	68.5%*	74.4%	31.6%*	67.3%	73.9%*	33.0%*	67.4%*	74.2%*
West	35.7%*	76.6%*	72.3%	35.6%*	76.5%*	72.3%*	35.2%*	76.1%*	72.3%*	36.2%*	75.9%*	72.0%*

Table C.11 shows the top 10 distinctive keywords by location for each source

Table C.11 Top 10 distinctive terms for location

Location	TwitterHealth	Google+Health	Drugs	Forums
Northeast	lupus<br>ovarian<br>fibromyalgia<br>pediatr<br>innovation<br>hospital<br>aarp<br>epidemic<br>gene	md<br>dry<br>lyme<br>ovarian<br>epidemiology<br>crohn<br>estrogen<br>morphin<br>novartis	iui<br>ivf<br>stabilized<br>gym<br>cancer<br>lamictal<br>distract<br>anxious<br>relaps	deb<br>antibodies<br>copd<br>bloodwork<br>gyn<br>af<br>dysfunction<br>ablation<br>tsh

	nurse	workout	celexa	bfn
Midwest	tumor<br>ibuprofen<br>colon<br>stomach<br>nap<br>medicaid<br>alcohol<br>tea<br>potassium<br>migraine	sharecare<br>valuim<br>diazepam<br>weed<br>unitedhealth<br>xanax<br>viagra<br>cigarette<br>seizure<br>chronic fatigue	dad<br>bleed<br>divorce<br>fibro<br>wellbutrin<br>allergy<br>cymbalta<br>boyfriend<br>ovulation<br>effect	pink<br>pelvic<br>diamox<br>vicodin<br>hysterectomy<br>estrogen<br>ob<br>medicaid<br>asthma<br>autism
South	hca<br>obamacare<br>uninsured<br>medicaid<br>aca<br>tension	ppo<br>januvia<br>disc<br>acne<br>nuvaring<br>myopia<br>coventry<br>caregiver<br>tragic<br>chiropractor	lyrica<br>cymbalta<br>sleepy<br>itch<br>fibromyalgia<br>kidney<br>child<br>wound<br>mania<br>father	billy<br>lyme<br>mtx<br>rheumy<br>cymbalta<br>cholesterol<br>codependent<br>endometriosis<br>medicaid<br>bc
West	kaiser permanente<br>lyme<br>remedy<br>autoimmune<br>rn<br>anxiety<br>cholesterole<br>heal<br>fibromyalgia<br>eczema	kaiser permanente<br>phenomenon<br>react<br>ldl<br>booster<br>conscious<br>antibody<br>sanofi<br>triglycerid<br>sooth	vitamin<br>groggy<br>lithium<br>alcohol<br>ptsd<br>aa<br>yoga<br>relapsing<br>brain<br>kidney	tinnitus<br>epilepsy<br>weed<br>dvt<br>vicodin<br>shunt<br>codependent<br>crohn<br>ptsd<br>hypothyroid

Table C.12 shows the top 5 drugs by location for each source

Table C.12 Top 5 distinctive drugs by location

**Location**	**TwitterHealth**	**Google+Health**	**Drugs**	**Forums**
Northeast	N/A	Diphosphonates<br>Humira<br>Epinephrine<br>Lyme Disease Vaccine<br>Sodium Chloride	Magnesium<br>Lupron<br>Klonopin<br>Lamictal<br>Vitamins	Diane<br>Sugars<br>Metformin<br>Carbohydrates<br>Smoke
Midwest	Ibuprofen<br>trivalent influenza vaccine	Ecstasy - drug<br>Aspartame<br>APT compounds<br>Cannabis substance<br>Viagra Pills	Abilify<br>Ritalin<br>Caffeine<br>Alcohols<br>Depakote	Immunoglobulins, Intravenous<br>Topamax<br>Diamox<br>Blood Glucose<br>human papilloma virus vaccine
South	N/A	Purified Protein Derivative of Tuberculin<br>Grit (substance)	Lyrica<br>Topamax<br>Cymbalta<br>Neurontin	Steroids<br>Topamax<br>Antibiotics<br>Cymbalta

		DC101 monoclonal antibody<br>Benzene<br>Aricept	Xanax	Lyrica
West	Viagra<br>Ergocalciferol	Taurine<br>Intermezzo<br>TimeLine Fluoride Releasing Resin<br>zolpidem<br>Antibodies	Lithium<br>Comfort brand of hydroxyethyl cellulose<br>Vitamins<br>Gluten<br>Amaze	Plaquenil<br>Alcohols<br>Hydrocortisone<br>Hormones<br>Smoke

C.5 Writing level

Table C.13 shows the sentiment by writing level for each source

Table C.13 Sentiment by reading level. O indicates ‘Objective’, P indicates ‘Positive’, and N indicates ‘Negative’. We put a “*” next to the values with high significance (p≤0.05) compared to the union of the other age groups.

	**TwitterHealth**			**Google+Health**			**Drugs**			**Forums**		
Writing level	O	P	N	O	P	N	O	P	N	O	P	N
0-5	N/A	N/A	N/A	86.3%*	5.9%*	6.3%	85.4%*	6.1%*	6.7%*	84.4%*	6.8%*	5.7%*
6-9	87.1%*	6.4%*	6.1%*	86.4%*	6.0%*	6.1%*	84.9%*	6.0%*	6.8%*	84.5%*	6.3%*	5.9%*
10-16	88.1%*	6.0%*	5.8%*	86.4%	5.8%*	6.3%	85.9%*	5.9%*	7.7%*	85.9%*	5.9%*	6.2%*

Table C.14 shows the top 10 distinctive terms by writing level for each source

Table C.14 Top 10 distinctive terms for writing level

**Writing level**	**TwitterHealth**	**Google+Health**	**Drugs**	**Forums**
0-5	N/A	ambien<br>nap<br>chicken pox<br>cry<br>grandma<br>daddy<br>pound<br>hurt	iui<br>ivf<br>cry<br>vent<br>carb	bfn<br>bfp<br>edd<br>gw<br>ttc<br>opk<br>af<br>pg<br>iui
6-9	kid<br>hiv<br>depress<br>aid<br>bipolar<br>breast<br>insomnia<br>asthma<br>vitamin<br>stroke	cardiologist<br>gabapentin<br>lyme<br>medic<br>ambulance<br>bp<br>doxycycline<br>neurologist<br>diazepam<br>hangover	vent<br>calorie<br>clomid<br>iui	diamox<br>rheumy<br>codependent<br>aa<br>ptsd<br>porn<br>bulli<br>miscarri<br>mtx<br>marriag

10-16	healthcare<br>diabetes<br>cure<br>vitamin<br>breast<br>heart<br>asthma<br>migraine<br>depress<br>hiv	tympanocentesis<br>myelin<br>cortex<br>encephalomyel<br>mediation<br>myalgic<br>lymphocytes<br>otitis<br>chloral<br>microbiology	physician<br>neurologist<br>medicin<br>remain<br>disc<br>oxycontin<br>narcotic<br>percocet<br>chronic<br>pharmacy	cardiac<br>physician<br>pd<br>diagnost<br>glucose<br>fda<br>surgical<br>neurology<br>urologist

Table C.15 shows the top 5 disorders by writing level for each source

Table C.15 Top 5 distinctive disorders for writing level

**Writing level**	**TwitterHealth**	**Google+Health**	**Drugs**	**Forums**
0-5	N/A	SHORT STATURE, AUDITORY CANAL ATRESIA, MANDIBULAR HYPOPLASIA, SKELETAL ABNORMALITIES<br>Insomnia Adverse Event<br>Chronic obstructive airway disease<br>hiv-infection/aids<br>Cough Adverse Event	N/A	DERMATITIS HERPETIFORMIS, FAMILIAL
6-9	Acquired Immunodeficiency Syndrome<br>Depressed mood<br>Malignant neoplasm of breast<br>HIV Seropositivity<br>Headache	Gait, Stumbling<br>Van der Woude syndrome<br>Tension<br>Feeling hopeless<br>Seizures	N/A	N/A
10-16	Presenile dementia<br>Diabetes Mellitus<br>Obesity<br>Primary Malignant Neoplasm<br>Malignant neoplasm of breast	Polycystic Kidney Diseases<br>KIDNEY FAILURE<br>Cardiovascular Diseases<br>Cyst<br>Complication	chronic pain<br>Mental Suffering<br>Vomiting<br>Severe pain<br>Attention Deficit Hyperactivity Disorder<br>Dizziness Adverse Event	Asthma<br>Diabetes Mellitus<br>Fibromyalgia<br>Primary Malignant Neoplasm<br>Hypersensitivity

Table C.16 shows the top 5 drugs by writing level for each source

Table C.16 Top 5 distinctive drugs by writing level

**Writing**	**TwitterHealth**	**Google+Health**	**Drugs**	**Forums**

level				
0-5	N/A	Hydrocodone<br>coconut oil<br>Ambien<br>Penicillins<br>Ibuprofen	N/A	Smoke
6-9	N/A	Grit (substance)<br>Diazepam Pills<br>sapphire<br>Acai extract<br>Viagra Pills	Avonex<br>Gluten<br>Comfort brand of hydroxyethyl cellulose<br>Lupron<br>Loestrin	Diamox<br>Plaquenil<br>Synthroid<br>Vent<br>Relate - vinyl resin
10-16	Viagra<br>Vitamins	Pradaxa<br>Taurine<br>Aldosterone<br>Thioctic Acid<br>Bicarbonates	Seasonique<br>Keppra<br>Tramadol<br>Lisinopril<br>Dilaudid	Ergocalciferol<br>Generic Drugs<br>Adderall<br>Dietary Lead<br>Oxygen

Appendix D Statistical Significance Tests

Tables D.1 reports the p-values for Mann-Whitney U test for sentiment and emotion by gender

Table D.1 p-values for Mann-Whitney U test for sentiment and emotion by gender

		**TwitterHealth**	**Google+Health**	**Drug review websites**	**Health Web forums**
Male vs Female	Objective	< 0.001	< 0.001	< 0.001	< 0.001
	Positive	0.1526	0.2574	< 0.001	< 0.001
	Negative	< 0.001	0.1148	< 0.001	< 0.001
Male vs Female	Anger	0.6206	0.1127	< 0.001	< 0.001
	Trust	< 0.001	< 0.001	< 0.001	< 0.001
	Anticipation	< 0.001	< 0.001	< 0.001	< 0.001
	Fear	< 0.001	< 0.001	< 0.001	< 0.001
	Disgust	< 0.001	0.0035	0.0044	< 0.001
	Surprise	0.0429	0.4973	< 0.001	< 0.001

Tables D.2 reports the p-values for Mann-Whitney U test for sentiment and emotion by age

Table D.2 p-values for Mann-Whitney U test for sentiment and emotion by age

		**Google+Health**	**Drug review websites**	**Health Web forums**
0-17 vs others	Objective	N/A	< 0.001	< 0.001
	Positive		< 0.001	0.3312
	Negative		< 0.001	< 0.001
18-34 vs others	Objective	0.0183	< 0.001	< 0.001
	Positive	0.3166	< 0.001	< 0.001
	Negative	0.0111	< 0.001	< 0.001
35-44 vs others	Objective	0.5751	< 0.001	< 0.001
	Positive	0.3458	< 0.001	< 0.001

	Negative	0.3722	< 0.001	< 0.001
45-64 vs others	Objective	< 0.001	0.5333	< 0.001
	Positive	0.0168	0.1058	< 0.001
	Negative	< 0.001	< 0.001	0.8853
65+ vs others	Objective	N/A	< 0.001	< 0.001
	Positive		< 0.001	< 0.001
	Negative		< 0.001	< 0.001
0-17 vs others	Anger	N/A	< 0.001	< 0.001
	Trust		< 0.001	< 0.001
	Anticipation		< 0.001	0.2976
	Fear		< 0.001	0.0088
	Disgust		< 0.001	< 0.001
	Surprise		< 0.001	0.6659
18-34 vs others	Anger	0.7186	< 0.001	< 0.001
	Trust	0.3024	< 0.001	< 0.001
	Anticipation	0.3998	< 0.001	< 0.001
	Fear	0.4055	0.1198	< 0.001
	Disgust	0.9208	< 0.001	< 0.001
	Surprise	0.9824	0.6265	0.0063
35-44 vs 45-64	Anger	0.6535	< 0.001	< 0.001
	Trust	0.7524	< 0.001	0.4127
	Anticipation	0.571	< 0.001	0.099
	Fear	0.1041	< 0.001	0.0781
	Disgust	0.0966	< 0.001	< 0.001
	Surprise	0.7911	< 0.001	< 0.001
45-64 vs others	Anger	0.5302	0.0094	< 0.001
	Trust	0.0501	< 0.001	< 0.001
	Anticipation	0.0386	0.0303	< 0.001
	Fear	0.0028	< 0.001	< 0.001
	Disgust	0.0051	0.0965	< 0.001
	Surprise	0.2975	0.0014	< 0.001
65+ vs others	Anger	N/A	< 0.001	< 0.001
	Trust		0.0184	0.6189
	Anticipation		0.0721	< 0.001
	Fear		< 0.001	0.0069
	Disgust		< 0.001	< 0.001
	Surprise		0.0012	0.4014

Tables D.3 reports the p-values for Mann-Whitney U test for sentiment and emotion by ethnicity

Table D.3 p-values for Mann-Whitney U test for sentiment and emotion by ethnicity

		**TwitterHealth**	**Google+Health**
White vs others	Objective	< 0.001	< 0.001
	Positive	< 0.001	< 0.001
	Negative	< 0.001	0.0024
Black vs others	Objective	0.0213	0.6474
	Positive	0.0346	0.3881
	Negative	0.4003	0.8228
Asian vs others	Objective	< 0.001	0.3381
	Positive	< 0.001	0.2503

	Negative	< 0.001	0.0108
Hispanic vs others	Objective	< 0.001	< 0.001
	Positive	< 0.001	< 0.001
	Negative	< 0.001	< 0.001
White vs others	Anger	< 0.001	0.3709
	Trust	< 0.001	0.7219
	Anticipation	< 0.001	0.0077
	Fear	< 0.001	0.0403
	Disgust	< 0.001	< 0.001
	Surprise	0.0964	0.9445
Black vs others	Anger	0.0239	0.9919
	Trust	0.0144	0.2548
	Anticipation	0.2002	0.5542
	Fear	0.5739	0.657
	Disgust	0.1978	0.1336
	Surprise	0.371	0.7182
Asian vs others	Anger	0.2777	0.7456
	Trust	0.0022	0.3082
	Anticipation	0.0075	0.0011
	Fear	0.6778	< 0.001
	Disgust	0.2867	< 0.001
	Surprise	0.0329	0.219
Hispanic vs others	Anger	< 0.001	0.371
	Trust	< 0.001	0.5066
	Anticipation	< 0.001	0.8316
	Fear	< 0.001	0.2907
	Disgust	< 0.001	0.5534
	Surprise	0.6044	0.1955

Tables D.4 reports the p-values for Mann-Whitney U test for sentiment and emotion by location

Table D.4 p-values for Mann-Whitney U test for sentiment and emotion by location

		**TwitterHealth**	**Google+Health**	**Drug review websites**	**Health Web forums**
Northeast vs others	Objective	< 0.001	< 0.001	0.0209	< 0.001
	Positive	0.0552	< 0.001	0.2456	< 0.001
	Negative	< 0.001	0.0768	< 0.001	< 0.001
Midwest vs others	Objective	< 0.001	0.0110	0.2205	< 0.001
	Positive	< 0.001	< 0.001	0.6888	< 0.001
	Negative	< 0.001	< 0.001	0.0266	< 0.001
South vs others	Objective	< 0.001	0.0030	< 0.001	< 0.001
	Positive	< 0.001	0.0125	< 0.001	< 0.001
	Negative	0.1185	0.4987	< 0.001	< 0.001
West vs others	Objective	0.0575	0.0365	< 0.001	0.0184
	Positive	0.0011	0.0020	< 0.001	0.0237
	Negative	< 0.001	0.0097	< 0.001	< 0.001
Northeast vs others	Anger	0.8666	0.1693	0.0076	0.0031
	Trust	0.0977	0.1945	0.3644	< 0.001
	Anticipation	0.1811	0.1507	0.4603	< 0.001
	Fear	0.1977	0.0702	0.8297	< 0.001

	Disgust	0.8374	< 0.001	0.4614	0.4283
	Surprise	0.9005	0.4697	< 0.001	0.12
Midwest vs others	Anger	0.0055	< 0.001	0.4121	0.0841
	Trust	0.1176	0.0179	0.0391	0.1065
	Anticipation	0.0412	0.0018	0.0328	0.0877
	Fear	0.0496	< 0.001	0.0155	0.0021
	Disgust	0.9082	< 0.001	0.0537	< 0.001
	Surprise	< 0.001	< 0.001	0.6786	< 0.001
South vs others	Anger	< 0.001	0.7772	< 0.001	< 0.001
	Trust	< 0.001	< 0.001	0.0564	< 0.001
	Anticipation	0.0686	0.2427	0.0169	< 0.001
	Fear	0.0082	0.479	0.6377	0.0327
	Disgust	< 0.001	0.9454	0.0362	< 0.001
	Surprise	0.2023	0.2762	0.0341	< 0.001
West vs others	Anger	< 0.001	0.0233	< 0.001	< 0.001
	Trust	< 0.001	0.0288	< 0.001	< 0.001
	Anticipation	0.257	< 0.001	< 0.001	< 0.001
	Fear	< 0.001	0.0247	0.0066	< 0.001
	Disgust	< 0.001	0.0467	0.1162	< 0.001
	Surprise	< 0.001	0.0197	0.1744	< 0.001

Tables D.5 reports the p-values for Mann-Whitney U test for sentiment and emotion by writing level

Table D.5 p-values for Mann-Whitney U test for sentiment and emotion by writing level

		**TwitterHealth**	**Google+Health**	**Drug review websites**	**Health Web forums**
0-5 vs others	Objective	N/A	< 0.001	< 0.001	< 0.001
	Positive		< 0.001	< 0.001	< 0.001
	Negative		0.1253	< 0.001	< 0.001
6-9 vs others	Objective	N/A	0.0062	< 0.001	< 0.001
	Positive		< 0.001	< 0.001	< 0.001
	Negative		< 0.001	< 0.001	< 0.001
10-16 vs others	Objective	< 0.001	0.4491	< 0.001	< 0.001
	Positive	< 0.001	0.0024	< 0.001	< 0.001
	Negative	< 0.001	0.7774	< 0.001	< 0.001
0-5 vs others	Anger	N/A	< 0.001	< 0.001	< 0.001
	Trust		0.0291	< 0.001	< 0.001
	Anticipation		0.7091	< 0.001	< 0.001
	Fear		0.4088	< 0.001	< 0.001
	Disgust		0.5039	< 0.001	< 0.001
	Surprise		< 0.001	< 0.001	< 0.001
6-9 vs others	Anger	N/A	< 0.001	< 0.001	< 0.001
	Trust		0.0016	< 0.001	< 0.001
	Anticipation		0.6083	< 0.001	< 0.001
	Fear		0.0221	< 0.001	< 0.001
	Disgust		0.5539	< 0.001	< 0.001
	Surprise		< 0.001	< 0.001	< 0.001
10-16 vs others	Anger	< 0.001	< 0.001	< 0.001	< 0.001

	Trust	< 0.001	< 0.001	< 0.001	< 0.001
	Anticipation	< 0.001	0.5228	< 0.001	< 0.001
	Fear	< 0.001	0.0093	< 0.001	< 0.001
	Disgust	< 0.001	0.3806	< 0.001	< 0.001
	Surprise	< 0.001	< 0.001	< 0.001	< 0.001

References

1 Twitter. https://twitter.com/

2 Google+. https://plus.google.com/

3 Drugs.com | Prescription Drug Information, Interactions & Side Effects. http://www.drugs.com/. Archived at: http://www.webcitation.org/6W23lHwlt

4 Treatments: reviews of drugs, therapies and remedies by everyday people – DailyStrength. http://www.dailystrength.org/treatments. Archived at: http://www.webcitation.org/6W23nXoJa

5 WebMD Drugs & Treatments - Medical Information and user ratings on prescription drugs and over-the-counter (OTC) medications. http://www.webmd.com/drugs/index-drugs.aspx. Archived at: http://www.webcitation.org/6W23rKUfB

6 Medical Questions Answered - Drugs.com. http://www.drugs.com/answers/. Archived at: http://www.webcitation.org/6W23vDAi5

7 WebMD - Better information. Better health. http://www.webmd.com/. Archived at: http://www.webcitation.org/6W247u0HQ

8 Online Support Groups - DailyStrength. http://www.dailystrength.org/support-groups. Archived at: http://www.webcitation.org/6W23ztpRe
